# Mechanical Behavior of a Reinforced Hourglass Lattice Structure

**DOI:** 10.3390/ma19040777

**Published:** 2026-02-16

**Authors:** Chong Liu, Wen Yang, Baifeng Sha, Henghao Zhang, Yongzhao Hou, Cheng Zhong, Meixian Jiang, Yongqiang Ma

**Affiliations:** 1 School of Transportation and Vehicle Engineering, Shandong University of Technology, Zibo 255000, China; 18730664063@163.com (C.L.); 15620610837@163.com (B.S.); 18653315923@163.com (H.Z.); 2 Shandong Provincial Key Laboratory of Integrated Design and Intelligence for Renewable Energy Vehicles, Shandong University of Technology, Zibo 255000, China; 3 School of Materials Science and Engineering, Shandong University of Technology, Zibo 255000, China; 4 Yantai Glass Coating Micro-Nano Imprinting Technology Innovation Center, Conor Glass Science & Technology Co., Ltd, Yantai 265700, China; jiangmeixian@conorgt.com (M.J.); mayongqiang@conorgt.com (Y.M.)

**Keywords:** lattice sandwich structure, bio-inspired design, reinforced hourglass core, local buckling suppression, in-plane and out-of-plane compression

## Abstract

Inspired by the networked venation structures, a reinforced hourglass lattice structure is proposed to overcome the insufficient face–core interaction and premature face-sheet buckling that limit the compressive performance of conventional lattice sandwich structures. The reinforced hourglass lattice structure is fabricated using a cutting–interlocking assembly followed by vacuum brazing, enabling enhanced connectivity and increased effective contact area between the lattice core and the face sheets. Quasi-static in-plane and out-of-plane compression experiments, together with finite element simulations and theoretical analysis, are conducted to systematically investigate the compressive behavior of the reinforced hourglass lattice structure. The results demonstrate that the out-of-plane compressive strength of the reinforced hourglass lattice structure exhibits a pronounced dependence on relative density, increasing monotonically with increasing density. Under in-plane compression, comparative studies with conventional hourglass and pyramidal lattice structures reveal that the proposed reinforcement strategy significantly improves face–core load transfer and effectively suppresses local buckling of thin face sheets. As a consequence, the reinforced hourglass lattice structure exhibits higher initial stiffness, enhanced compressive strength, and superior structural stability. These findings indicate that reinforcing the reinforced hourglass core provides an effective design strategy for improving the compressive performance of lattice sandwich structures by strengthening face–core interaction and mitigating face-sheet buckling.

## 1. Introduction

Compared to traditional solid structures, lattice structures have attracted widespread attention in recent years in various fields such as aerospace [1], civil engineering [2], and transportation [3], due to their unique characteristics of being lightweight and design-flexible and having excellent high strength and stiffness. In order to meet the requirements of various fields for lightweight and high-strength structural performance, researchers have conducted extensive research to improve the performance of lattice structures.

Previous studies have proposed various lattice structures with excellent performance, including cubic lattice [4], Kagome lattice [5], body-centered cubic (SC-BCC) [6] topologies, and zero Poisson’s ratio hybrid lattices [7]. Yang et al. [8] conducted experimental and finite element analysis to compare the energy absorption capability and deformation modes of three different structures. The results showed that the Kelvin lattice structure [9] exhibited optimal overall mechanical performance. Moreover, FCC [10] and Octet [11] lattice structures outperformed the Kelvin lattice in terms of anisotropy. However, most lattice structures in practical applications face issues such as insufficient contact strength between the lattice and the panel, complex manufacturing processes, and susceptibility to buckling failure. Longhitano et al. [12] investigated the mechanical behavior and fracture characteristics of additively manufactured Ti-6Al-4V lattice structures and demonstrated that finite element analysis (FEA) provides a more reliable tool for predicting fracture behavior than classical analytical criteria.

To address these issues, Zhao et al. [13] studied the compression behavior and failure modes of pyramid-shaped lattices under both node unconstrained and node-constrained conditions. The study found that the failure mode of the node-constrained structure was primarily due to pillar buckling and fracture, allowing the lattice to maximize the material’s performance, whereas, in the node-unconstrained case, joint displacement failure occurred during compression, leading to delamination and cracking. Tohid et al. [14] significantly improved the compression performance of the panel by reinforcing the core-panel node, solving the early failure problem of the fiber core panel and central node. Li et al. [15] analyzed the impact of local defects on the mechanical and creep properties of pyramid-shaped lattice panel structures. Korshunova et al. [16] applied an embedded numerical framework to better simulate the discrepancies between numerical simulation results and experimental results caused by defects in metal additive manufacturing, effectively simulating the mechanical behavior of lattice structures. Ge et al. [17] provided adequate support by increasing the bonding area between the pyramid lattice and the covering panel, ensuring the continuity between the lattice and the panel. Dastan et al. [18] significantly improved the compression performance of the panel with minimal weight increase by reinforcing the core-panel node. Xu et al. [19] compared the fracture toughness of different material types in 3D octet lattice structures. Emami et al. [20] fabricated ceramic Kelvin lattice structures and found that the shear strength of these ceramic lattices was pressure-sensitive, providing better performance than traditional materials.

Moreover, the innovative design of lattice structures requires further exploration to meet higher performance demands. The development of advanced manufacturing processes, such as additive manufacturing, provides an important foundation for designing complex structures. Yang et al. [21] conducted out-of-plane and in-plane compression and shear tests on metal pyramid lattice structures. Under in-plane compression, panel plastic yielding and lattice deformation led to a decrease in support capacity. To optimize the performance of lattice structures, researchers have designed various new structures, including flexible 3D porous lattices [22] and triply periodic minimal surface (TPMS) structures [23]. Zhu et al. [24] conducted comparative studies based on lattice structures and found that sandwich structures with plate lattice cores could achieve uniform strain field distribution, thereby more effectively bearing loads. Wu et al. [25] and Dalakoti et al. [26] proposed structures combining plates with hollow lattices, enhancing the interaction between them to alter the deformation behavior, thus improving fracture stress, material utilization, and energy absorption capacity. Inspired by triply periodic minimal surfaces (TPMSs), Christian et al. [27] introduced fileted geometries into metallic cubic lattice structures. Their results demonstrated that replacing sharp node intersections with smooth, bio-inspired filets induces a favorable triaxial stress state at the joints. This geometric optimization significantly enhances the strength-to-weight ratio, outperforming conventional lattice designs with sharp corners.

Ghasemi et al. [28] compared the performance of gradient and uniform lattice sandwich panels under impact loads and found that, although the uniform lattice structure had higher strength, the gradient lattice structure absorbed more energy due to greater plastic deformation. Zhang et al. [29] investigated systematic design strategies for enhancing the toughness of lattice sandwich structures and established a corresponding toughening model based on structural failure modes. Their results revealed a non-monotonic relationship between strut diameter and impact toughness. Hu et al. [30] designed foam-filled double-layer lattice sandwich panels and found that the interaction between the core rods and the foam effectively suppressed lateral shear and bending deformation of the lattice structure. Wang et al. [31] and Feng et al. [32] achieved isotropic lattice design and anisotropy control strategies by adjusting shape parameters. Zhang et al. [33], Deng et al. [34], and Dong et al. [35] combined octet node lattice basic units with other basic cells to design topologies with better performance than octet node lattices. Previous studies have predominantly focused on improving the lattice structures themselves, while comparatively limited attention has been paid to the role of the lattice–face sheet connection in enhancing the overall mechanical performance of the structure.

Unlike traditional node reinforcement strategies that only focus on preventing failure of internal support rods, this paper introduces a topology-expanded panel-core interface to construct an enhanced hourglass structure. Through simulation analysis and experimental verification, the in-plane compression performance and out-of-plane compression performance of the enhanced hourglass lattice structure are studied, and comparative analysis is conducted with traditional hourglass lattice structures and pyramid lattice structures.

## 2. Reinforced Hourglass Lattice Structure Design and Fabrication

### 2.1. Design of Reinforced Hourglass Lattice Structure

The design concept of the reinforced hourglass lattice structure is inspired by networked venation systems commonly observed in natural biological materials. By locally reinforcing the lattice core, the effective contact area between the face sheets and the core is increased, which is expected to enhance the local buckling resistance of the face sheets and improve the deformation resistance of the load-bearing struts under compressive loading.

**Figure 1:**
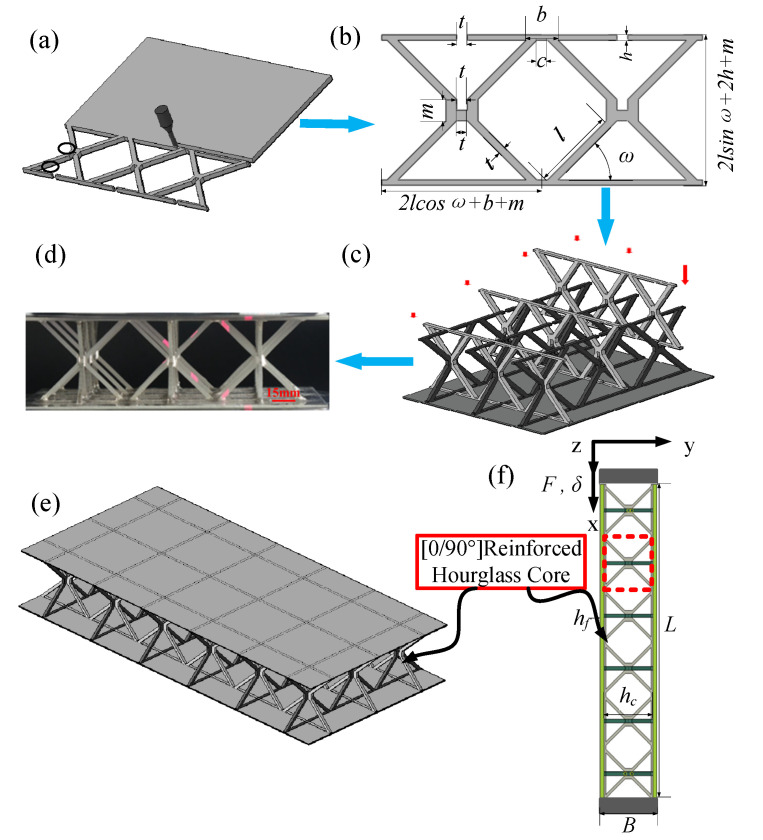
Reinforced hourglass lattice structure fabrication and assembly method schematic diagram: (**a**) WEDM; (**b**) reinforced hourglass lattice structure (2D); (**c**,**d**) 3 × 3 lattice model; and (**e**,**f**) 3 × 6 lattice model.

Figure 1a–c illustrate the schematic configuration of the reinforced hourglass lattice structure. In this design, the upper and lower segments of the original hourglass struts that are in contact with the face sheets are locally reinforced, leading to an enlarged contact area at the face–core interface and improved structural stability under external loading. Two-dimensional unit cells are connected through the upper and lower void regions to form a three-dimensional unit cell of the reinforced hourglass lattice structure, as shown in Figure 1c. The corresponding geometric parameters of the reinforced hourglass lattice structure are summarized in Table 1. For out-of-plane compression tests, a 3 × 3 array of reinforced hourglass unit cells was assembled to form the sandwich structure. The lattice core was bonded to the top and bottom face sheets, and the corresponding fabricated specimen is shown in Figure 1e. For in-plane compression tests, a 3 × 6 reinforced hourglass lattice structure array was employed. Similarly, the lattice core was integrated with the top and bottom face sheets to form the reinforced hourglass lattice structure, as shown in Figure 1e,f.

When the relative density was set to 1.007%, the face sheets had in-plane dimensions of 192 × 96 mm, and the face-sheet thickness was fixed at H_f_ = 0.4 mm. The relatively thin face sheets were intentionally selected to more clearly reveal the enhancement in local buckling resistance provided by the reinforced lattice core. By keeping the node size of the core struts constant and varying the strut length, reinforced hourglass lattice structures with relative densities of 1.007%, 1.76%, and 2.48% were fabricated. The relative densities were calculated according to Equation (1), and the corresponding reinforced strut heights and aspect ratios are listed in Table 2. Through appropriate combinations of lattice geometric parameters, reinforced hourglass lattice structures with different relative densities were designed to achieve a range of targeted mechanical performances.

(1) $$\bar{\rho }=\;\frac{8lt^{2}+\;4\left(2lcos\omega \;+\;b\;+\;m\right)ht\;+\;2\left(2\sqrt{2}\;+\;1\right)mt^{2}+\;4t^{3}}{{\left(2lcos\omega \;+\;b\;+\;m\right)}^{2}\left(2lsin\omega \;+\;2h\;+\;m\right)}$$

### 2.2. Lattice Fabrication

The reinforced hourglass lattice structure used for the out-of-plane compression tests consisted of a 3 × 3 reinforced hourglass core bonded to the top and bottom face sheets, as shown in Figure 1d,e. The fabrication procedure of the reinforced hourglass lattice structure is schematically illustrated in Figure 1. The lattice structures were manufactured using a cutting–insertion–assembly–brazing process. Initially, reinforced hourglass lattice structure components were cut from a 1.4 mm thick 304 stainless steel plate using Wire Electrical Discharge Machining (WEDM). High dimensional accuracy was required in the assembly regions, as highlighted by the black-marked areas in Figure 1a, since the machining precision in these regions directly influences the geometric continuity and mechanical integrity of the assembled three-dimensional reinforced lattice structure.

Subsequently, the cut lattice components were assembled through orthogonal insertion at 0° and 90°, forming the three-dimensional reinforced hourglass core, as shown in Figure 1b,c. Nicrobraze 31 filler metal was then applied to the contact regions between the lattice nodes, the reinforced struts, and the top and bottom face sheets. A small external pressure was applied to ensure intimate contact at the joints. The assembled reinforced hourglass lattice structures were placed in a high-temperature vacuum brazing furnace for joining. During the brazing process, the temperature was first increased to 950 °C at a heating rate of 15 °C/min and held for 30–60 min to ensure sufficient preheating of the filler metal. The temperature was subsequently raised to 1050 °C at a rate of 20 °C/min, and the structure was held under a vacuum pressure of 2 × $10^{-2}$ Pa for 6–10 min. Upon reaching the melting temperature of the filler metal, the molten brazing alloy infiltrated the interfacial gaps between the metallic components, resulting in metallurgical bonding. After brazing, the furnace heating was turned off, and the specimens were allowed to cool naturally to room temperature, during which the filler metal solidified and formed high-strength joints.

The enlarged face–core contact region introduced by the reinforced hourglass lattice design further reduces sensitivity to manufacturing defects, thereby improving structural reliability and defect tolerance. In addition, the thermal cycling involved in the brazing process is considered to have an annealing effect, which helps relieve the residual stresses induced by Wire Electrical Discharge Machining. However, since the present study focuses on macroscopic structural performance, the detailed effects of brazing-induced defects and residual stresses were not explicitly modeled.

### 2.3. Base Material Properties (Tensile and Compressive Response of Material)

**Figure 2:**
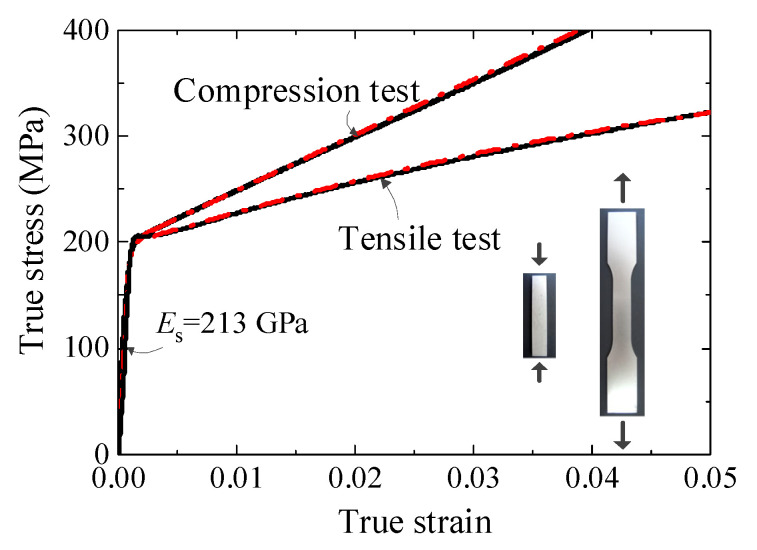
Tensile and compressive true stress–true strain curves of 304 stainless steel.

Both the reinforced hourglass lattice structure and the face sheets were fabricated from 304 stainless steel. To characterize the mechanical behavior of the base material, 304 stainless steel plates were subjected to uniaxial tensile and compressive tests. Prior to testing, all specimens were heat-treated in a furnace to eliminate residual stresses introduced during machining. The tensile and compression tests were conducted in accordance with ASTM E8-01 [36] and ASTM E9-89a [37] standards, respectively. Three replicate specimens were tested for each loading condition to ensure the repeatability and reliability of the measured responses. Figure 2 presents the true stress–true strain curves obtained from the tensile and compressive tests of the 304 stainless steel plate, with the experimental data shown as solid black lines. For subsequent theoretical analysis and finite element simulations, the experimental stress–strain responses under tension and compression were separately fitted using a modified Ramberg–Osgood [38] (R–O) constitutive model. The fitted curves are indicated by the red dashed lines in Figure 2 and provide the material parameters required for the numerical modeling of the reinforced hourglass lattice structures.

### 2.4. Finite Element Model

To evaluate the mechanical performance of the reinforced hourglass lattice structures, finite element simulations were conducted in conjunction with experimental tests. All numerical analyses were performed using the commercial finite element software ABAQUS 2020 which enables the consideration of material nonlinearity, geometric nonlinearity, and contact nonlinearity, thereby allowing accurate prediction of the nonlinear deformation and failure behavior of reinforced hourglass lattice structures.

Under out-of-plane compression conditions, the contact between the top and bottom panels and the reinforced hourglass core was modeled using the “tie” connection method to ensure that the contact settings between the panel and lattice were the same as in the experiments. The use of the “tie” connection was motivated by experimental observations from both in-plane and out-of-plane compression tests, in which the failure mechanisms were primarily dominated by strut buckling or face-sheet yielding. No pronounced delamination or crack initiation was observed at the joint regions, indicating that the overall mechanical response can be reasonably captured using a “tie” constraint. Since this study mainly focuses on the macroscopic structural performance rather than the microscopic fracture behavior of the brazed joints, the “tie” constraint provides a computationally efficient and sufficiently accurate approximation. However, for studies in which the joining process has a significant influence on the experimental response, potential residual stresses induced by the manufacturing process should be taken into account. The freedom of the bottom surface of the bottom panel was constrained in all six directions to implement a fully fixed boundary condition. The interaction between the overall structure was set as tangential penalty contact with a static friction coefficient of 0.2 and normal hard contact. For the loading method, the upper surface of the top panel was coupled with a reference point, and the loading rate for the reference point was set to 5 × $10^{-4}s^{-1}$ (the same as in the experiments), with the ABAQUS/static analysis step used for the simulation. Both the panel and the reinforced hourglass lattice structure were modeled using C3D8R solid elements.

**Figure 3:**
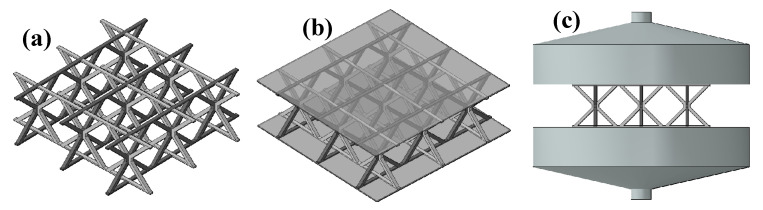
Out-of-plane compression model: (**a**) 3 × 3 reinforced hourglass lattice structure model; (**b**) complete 3 × 3 reinforced hourglass structure model; and (**c**) overall compression model.

For out-of-plane compression, the face sheets exhibit relatively small deformations; therefore, a mesh size of 0.8 mm was adopted for the face sheets, while a finer mesh size of 0.4 mm was used for the lattice core to accurately capture local deformation and buckling behavior. For in-of-plane compression simulations, where both the face sheets and lattice core undergo large deformations, a uniform mesh size of 0.4 mm was applied to both components. Mesh convergence studies were conducted to ensure that the numerical results were insensitive to the chosen element sizes. The final finite element models for the out-of-plane and in-plane compression simulations are shown in Figure 1e,f and Figure 3 respectively. The material properties of 304 stainless steel adopted in the simulations were obtained from the experimental characterization described in Section 2.3.

## 3. Mechanical Properties of Reinforced Hourglass Lattice Structure

### 3.1. Out-of-Plane Compression

To investigate the out-of-plane compressive response of the reinforced hourglass lattice structures with different relative densities, quasi-static compression tests were performed on specimens with three representative relative densities (three repeated samples were prepared for each specimen configuration). The loading was applied normal to the top face sheet at a nominal strain rate of 5 × $10^{-4}s^{-1}$.

**Figure 4:**
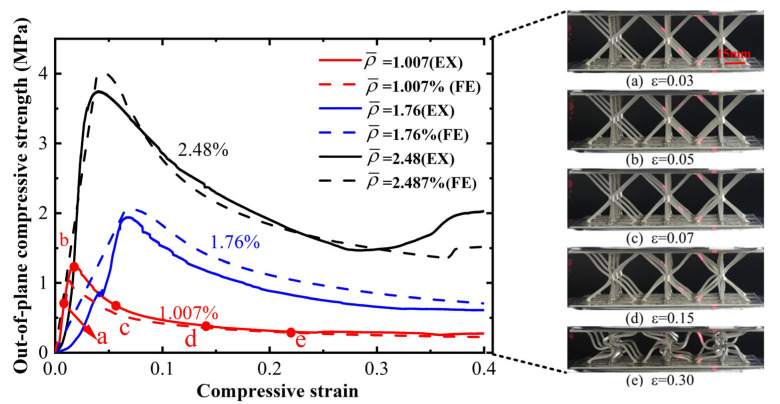
Stress–strain curves of reinforced hourglass lattice structures with different $\bar{\rho }$.

As shown in Figure 4, the out-of-plane compression response of the reinforced hourglass lattice structures can be generally divided into an initial elastic stage followed by a yielding stage. For the structure with the highest relative density of 2.48%, a distinct densification stage is observed at large strains. With increasing relative density, both the elastic stiffness and the peak compressive strength increase monotonically. This trend indicates that a denser lattice configuration provides enhanced resistance to out-of-plane deformation. In addition, the area enclosed by the stress–strain curve increases with relative density, demonstrating an improved energy absorption capability under out-of-plane compression. It is noted that the maximum compressive strength for the three reinforced hourglass lattice structures does not occur at the same strain level. For the lowest relative density of 1.007%, the structure exhibits relatively low stiffness and enters the yielding regime at a small strain, resulting in an early peak in compressive strength, and Figure 4 illustrates the corresponding deformation processes (a–e) at typical points along the load–strain curve. In contrast, the structure with a relative density of 2.48% shows significantly higher stiffness, which delays the onset of yielding and shifts the peak strength to a larger strain. The structure with an intermediate relative density of 1.76% (the blue curve) is in a transitional zone between elastic buckling and plastic yielding before reaching the peak load. In this regime, the structure undergoes a coupled elastic–plastic deformation and a progressive buckling process. As the load increases, this manifests as the pronounced non-linear curvature observed prior to the peak. This mechanism facilitates a gradual accumulation of deformation, ultimately resulting in a more uniform deformation mode and a delayed transition into the yielding stage compared to the other configurations. By comparing the experimental and simulated stress–strain curves as well as the corresponding deformation modes for the three relative densities, a good agreement between the two is observed, validating the reliability of the finite element model in accurately capturing the out-of-plane compressive behavior of the reinforced hourglass lattice structures.

From Table 3, it can be observed that the reinforced hourglass lattice structure with a relative density of 1.007% is in the elastic deformation stage during the initial loading phase (strain range of 0.00–0.02). The stress distribution inside the lattice can be visualized through the simulation deformation diagram. At this stage, the stress concentration in the hourglass lattice structure is significant, especially in the eight vertical rods, which become the primary supporting elements during this phase and experience slight deformation. However, no significant stress concentration occurs in the areas where the reinforced structure contacts the panel. It is noteworthy that a stress variation appears at the intersection between the reinforced structure and the hourglass lattice structure, indicating that the reinforced structure plays a strengthening role in the overall elastic deformation process of the structure. As the loading continues, the structure’s stress gradually increases and reaches its peak. When the stress exceeds the material’s elastic limit, the structure begins to enter the plastic deformation stage, characterized by a gradual decrease in stress accompanied by irreversible plastic deformation. The yield stage typically occurs at strains between 0.02 and 0.4. From the deformation diagram, it can be observed that the rods start to bend or buckle, and the overall eight rods exhibit an X-shaped twisting deformation. When the strain exceeds 0.3 for reinforced hourglass lattice structure of 2.48%, the structure enters the densification stage. During this stage, the overall volume of the reinforced hourglass lattice structure gradually decreases, and the gaps between the rods are gradually filled, leading to a reduction in internal voids. As the strain increases, the stress in the lattice gradually increases again, indicating that the structure has entered the densification stage.

By comparing the deformation diagrams of the reinforced hourglass lattice structures with three different relative densities in Table 3, it can be observed that the differences in the deformation modes of the reinforced hourglass lattice structure under different densities are not significant. Further observation of the stress distribution at the connection points between the reinforced rods and the hourglass rods reveals that as the relative density increases, the region where the reinforced rods bear stress gradually expands. In the reinforced hourglass lattice structure with a low relative density, the aspect ratio of the rods is high, which means that the rods are more flexible and prone to buckling. During out-of-plane compression, when the stress has not reached the yield limit, the structure experiences instability and deformation, causing the reinforced rods to fail to effectively bear the stress. Therefore, under low relative density conditions, the stress distribution area at the connection point between the reinforced rods and the hourglass rods in the deformation diagram is relatively small. However, as the relative density increases, the aspect ratio of the rods decreases, resulting in an increased critical yield load for the hourglass rods. This allows the reinforced structure to better share the stress, leading to greater stability during out-of-plane compression and effectively resisting buckling and deformation.

**Figure 5:**
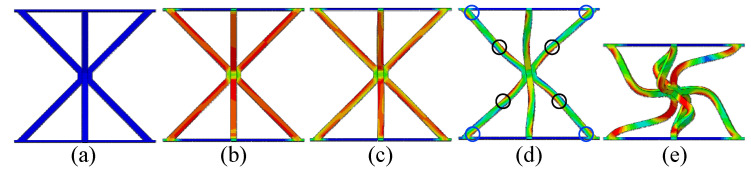
Deformation modes of the reinforced hourglass unit cell: (**a**) Initial undeformed configuration; (**b**) Elastic deformation stage; (**c**) Progressive buckling of struts; (**d**) Plastic hinge formation and rotation; and (**e**) Buckling-induced collapse mode.

The reinforced hourglass lattice structure is constructed by periodically arraying reinforced hourglass unit cells, and a proportional relationship exists between the plateau stresses of the lattice and those of the corresponding unit cell. Therefore, the plateau stress of the reinforced hourglass lattice can be determined by first evaluating the plateau stress of a single reinforced hourglass unit cell. The plateau stress is an important indicator for evaluating the compressive performance of lattice structures. To derive the analytical expression, several simplifying assumptions are adopted: the material is considered rigid–perfectly plastic. The collapse mechanism is dominated by strut bending, while the contributions of axial compression and shear forces to plastic dissipation are assumed negligible; and the lattice nodes are treated as rigid joints, with deformation localized at the plastic hinges. These assumptions are most appropriate for lattice structures with low relative densities and slender struts. Under quasi-static out-of-plane compression, when the bending moment in the struts reaches the fully plastic bending moment, the unit cell enters the plastic collapse regime. The deformation process of the reinforced hourglass unit cell is illustrated in Figure 5.

Based on the principle of energy conservation, the external work performed by the compressive force is equal to the internal plastic dissipation associated with the formation of plastic hinges and their corresponding rotation angles (i.e., the rotations of all plastic hinges), Equation (2) is obtained.

(2) $$8M_{p}\theta_{0}\;+\;8M_{p}\theta_{1}\;=\;F_{P}∆y$$

(3) $$M_{p}=\;\sigma_{s}\frac{t^{3}}{4}$$

(4) $$∆y\;=\;2L\left(sinw\;-\;sin\theta_{1}\right)$$

*M_p_* represents the plastic bending moment, which is calculated according to Equation (3). $\sigma_{s}$ is the material stress, $\theta_{0}$ and $\theta_{1}$ are the deformation angles of the reinforced hourglass unit cell during compression, ∆*y* is the axial displacement, the corresponding analytical expression derived from the structural deformation process is given by Equation (4) and $F_{p}$ is the compressive force corresponding to the collapse of the reinforced hourglass unit cell.

By equating the external work to the internal plastic dissipation and substituting the expressions for the plastic bending moment together with the geometric relationships of the unit cell, the theoretical expression for the plateau stress of the reinforced hourglass core is obtained as follows:

(5) $$\sigma_{p}=\;\frac{\left(8\theta_{0}+\;8\theta_{1}\right)}{8L\left(sinw\;-\;sin\theta_{1}\right){\left(2Lcosw\;+\;b\;+\;m\right)}^{2}}\sigma_{s}t^{3}$$

Using Equation (5), the theoretical plateau stresses of the reinforced hourglass lattice structures with relative densities of 1.007%, 1.76%, and 2.48% were calculated. The corresponding theoretical predictions (TPs), finite element (FE) results, and experimental (EX) measurements are summarized in Table 4.

### 3.2. In-Plane Compression

The in-plane compression specimens of the reinforced hourglass lattice structures investigated in this section had a span length of 185 mm and a width of 100 mm. All in-plane compression tests were conducted at a constant loading rate of 0.5 mm/min. To evaluate the in-plane compressive performance of the reinforced hourglass lattice structure, comparative tests were performed against the conventional hourglass lattice structure and the pyramidal lattice structures. Three repeated experiments were conducted for each structural configuration to account for possible fabrication imperfections.

**Figure 6:**
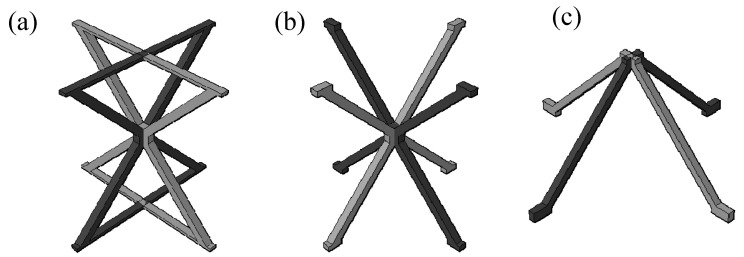
Geometric configurations of the different lattice core: (**a**) reinforced hourglass unit cell, (**b**) conventional hourglass unit cell, and (**c**) pyramidal unit cell.

The three structures are illustrated in Figure 6. The pyramidal unit cell consists of two inverted V-shaped struts converging at a central node. The hourglass unit cell can be geometrically derived by mirroring the pyramidal unit cell about the horizontal plane passing through the central node. The reinforced hourglass unit cell preserves the core topology of the conventional hourglass lattice, while horizontal reinforcing segments are incorporated at the top and bottom of the struts, replacing point contacts with enlarged planar contacts. Considering the differences in topology among the three lattice structures, slight variations in relative density are inevitable. To ensure comparability among the three designs, as shown in Table 5, the relative density of the reinforced hourglass lattice ($\bar{\rho }$ = 1.007%) was deliberately designed to be slightly lower than that of the conventional hourglass lattice ($\bar{\rho }$ = 1.15%).

**Figure 7:**
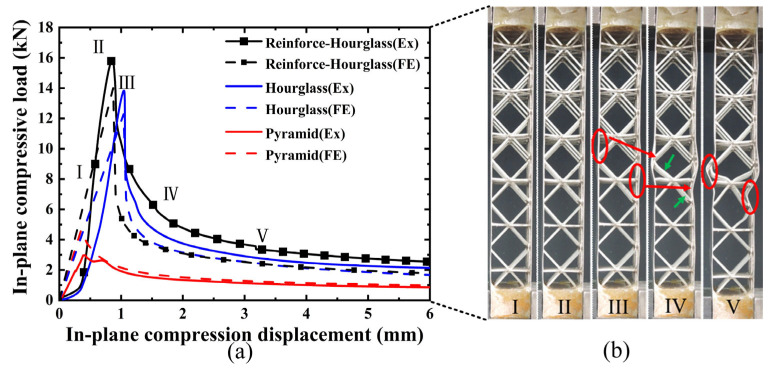
In-plane compressive behavior of different lattice structures: (**a**) load–displacement curve (I: Elastic response stage, II: Critical limit state (Peak load), III: Initial Facesheet Buckling, IV: Coupled Strut-Panel Deformation, and V: Severe Panel Collapse), and (**b**) typical deformation modes.

As shown in Figure 7a, the three structures demonstrate similar fluctuation trends in their load–displacement responses under in-plane compression. The response initially shows a linear elastic regime, followed by yielding accompanied by mild strain hardening. With further increase in displacement, elastic or inelastic local buckling of the face sheets occurs, leading to the attainment of the peak load. Beyond the peak load, softening of the reinforced hourglass lattice structure is observed, and the load gradually decreases. The results indicate that the dominant failure mode of all specimens is local buckling of the face sheets. However, owing to the differences in the lattice core configurations, the extent and distribution of deformation within the reinforced hourglass lattice structures vary significantly. The reinforced hourglass lattice structure exhibits a significantly higher maximum in-plane compressive load than the other two configurations. Specifically, the peak in-plane compressive load of the reinforced hourglass lattice structure is approximately 1.144 times that of the hourglass lattice structure and 5.345 times that of the pyramidal lattice. W is defined as the area enclosed by the load–displacement curve, representing the energy absorption capacity of the three structures under out-of-plane compression. The energy absorption of the reinforced hourglass lattice structure is approximately 1.3 and 3.4 times that of the hourglass lattice structure and the pyramidal lattice structure, respectively. The detailed geometric dimensions and mechanical performance parameters of the three lattice structures are summarized in Table 5.

The experimentally observed deformation modes, as presented in Figure 7b. After reaching the peak load, subsequent loading triggers initial face-sheet buckling, as indicated by the red circles at III in Figure 7b. Due to the reinforcement design at the interface between the reinforced hourglass lattice structure and the face-sheets, effective support is provided to inhibit face-sheet buckling, as highlighted by the green arrows at IV. With continued loading, the softening of the reinforcement struts and pronounced face-sheet buckling occur, as shown in the red circles at V. Owing to the restricted degrees of freedom at the bottom relative to the top, the in-plane compressive load is primarily transferred to the left and right face sheets. The load-bearing struts in the lattice redistribute the applied in-plane compressive load through mutual support and structural connectivity, resulting in an increased bending stiffness of the overall structure and a reduced susceptibility to global deformation. As illustrated in Table 6, the failure of hourglass and pyramid lattices is triggered by premature face-sheet buckling. The reinforced design effectively reduces the unsupported span of the face sheet. According to plate buckling theory, stability increases quadratically as this span decreases. By suppressing local buckling, the reinforcement allows the face sheets to maintain their load-carrying capacity for longer. This enables a more efficient load transfer into the lattice core, leading to the “distributed homogeneous stress state” observed in the FEA results. But the reinforcement strategy does not fundamentally alter the global geometry or slenderness ratio of the struts. Therefore, the observed performance improvement can be primarily attributed to the modification of the face–core interaction and load transfer mechanism. A comparison of the key parameters of the three structures in Table 5 indicates that the reinforced hourglass lattice demonstrates markedly higher specific strength and energy absorption performance. Although its relative density is lower than that of the conventional hourglass lattice, the peak load of the reinforced design is increased by 13.5%. Furthermore, relative to the conical lattice, the proposed structure exhibits approximately five times higher strength and 3.4 times greater energy absorption capacity.

By comparing the load–displacement responses and deformation modes (Table 6) of the reinforced hourglass lattice structure and the conventional hourglass lattice structure under identical in-plane compression conditions, pronounced differences in mechanical performance are observed. For the unreinforced hourglass lattice structure, the face–core interface lacks additional connections and local support, resulting in insufficient coordination between the face sheets and the lattice core. Consequently, the applied in-plane load cannot be effectively transferred from the face sheets to the lattice core, leading to pronounced stress concentrations in the central region of the face sheets. These localized stress concentrations promote premature local buckling of the face sheets, thereby compromising the overall structural stability. Therefore, the load increases gradually during the initial loading stage. With increasing displacement, the structure rapidly enters yielding and instability regimes, resulting in load degradation and a reduced load-carrying capacity. Both the initial stiffness and the ultimate in-plane compressive strength of the unreinforced hourglass lattice structure are markedly lower than those of the reinforced configuration. For the pyramidal lattice structure, similar to the unreinforced hourglass lattice structure, the number of effective physical connections between the lattice core and the face sheets is limited, with overly large nodal spacing. As a result, the face sheets of the pyramidal structure yield at an early stage, leading to insufficient overall load-bearing capacity. The load–displacement response clearly indicates that the pyramidal lattice exhibits a substantially lower in-plane compressive strength than both the hourglass lattice and the reinforced hourglass lattice structure. This inferior performance can be primarily attributed to the lack of coordinated deformation between the face sheets and the pyramidal lattice core. Due to the absence of effective load transfer paths, the applied in-plane load is predominantly carried by the face sheets, resulting in pronounced stress concentrations and premature yielding, particularly in the central region of the specimen. Meanwhile, the pyramidal lattice core undergoes only limited deformation and fails to provide sufficient structural support, thereby contributing minimally to the overall load-carrying capacity. Consequently, the pyramidal lattice structure exhibits a low initial stiffness and poor in-plane compressive stability.

In summary, the reinforced hourglass lattice structure significantly enhances the in-plane compressive performance and structural stability of the structure by improving face–core interaction and promoting efficient load transfer. In contrast, both the unreinforced hourglass lattice structure and the pyramidal lattice suffer from inadequate face–core coordination, resulting in lower initial stiffness and reduced in-plane compressive strength. These results demonstrate the clear mechanical advantage of the reinforced hourglass lattice structure over conventional hourglass and pyramidal lattice configurations under in-plane compression.

## 4. Conclusions

This study proposed and systematically evaluated a bio-inspired reinforced hourglass lattice structure designed to overcome the premature face-sheet buckling limitations of conventional sandwich cores. Through a combination of “cutting–interlocking–brazing” fabrication, experimental testing, theoretical modeling, and finite element analysis, the following conclusions are drawn:

1. The reinforced hourglass topology alters the face–core interaction mechanics. Unlike conventional point-contact lattices (hourglass and pyramidal structure) where the unsupported span is large, the proposed reinforcement expands the nodal contact area, significantly reducing the effective buckling span of the face sheets. By suppressing local buckling, the reinforcement allows the face sheets to maintain their load-carrying capacity for longer.
2. The out-of-plane strength of the reinforced hourglass lattice exhibits a strong dependence on relative density. The failure mechanism transitions from elastic buckling in low-density structures to plastic yielding in high-density configurations. A theoretical model based on the plastic hinge mechanism was developed, which accurately predicts the plateau stress for bending-dominated deformation modes.
3. In comparative studies, the reinforced hourglass lattice demonstrated superior specific strength and energy absorption. Despite having a lower relative density than the conventional hourglass lattice, the reinforced structure achieved a 13.5% higher peak load. Compared to the pyramidal lattice, the proposed design exhibited a 5-fold increase in strength and a 3.4-fold increase in energy absorption.

Although the present study provides valuable insights, it is limited to quasi-static loading conditions and flat-plate structures fabricated from ductile stainless steel. Future work will focus on: (1) evaluating the strain-rate sensitivity and impact resistance of the proposed structure under dynamic loading conditions; (2) investigating the scalability and applicability of the interlocking assembly approach to complex curved surfaces; and (3) extending the design concept of enhancing the effective contact area between the face sheets and the lattice core to other structural configurations.

## Figures and Tables

**Table 1:** Geometric dimensions of the reinforced hourglass lattice structure.

Geometric Dimensions	*t*	*w*	*b*	*c*	*m*	*h*	*ω*
Reinforced Hourglass (mm)	1.4	1.4	4.5	3.48	1.5	0.50	45°

**Table 2:** Reinforced hourglass lattice structure parameters and out-of-plane compressive strength.

Core Type		Aspect Ratio (*t/l*)	Core Height (*h_c_* mm)	$\bar{\rho }$ (%)	Compressive Strength (MPa)
Reinforced Hourglass	A	0.073	30	1.007	1.36
	B	0.107	22	1.76	2.25
	C	0.135	18.5	2.48	3.74

**Table 3:** Typical deformation modes of reinforced hourglass lattice structures at three $\bar{\rho }$ (simulation).

$\bar{\rho }$	1.007%		1.76%		2.48%	
Strain	0.007	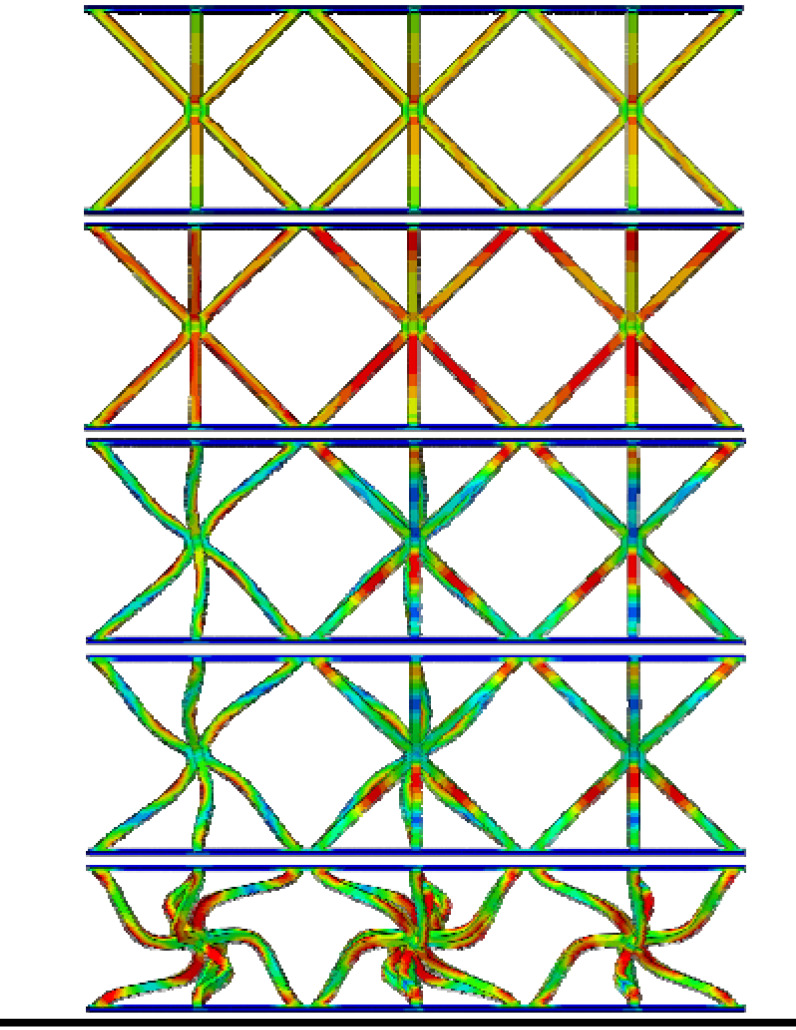	0.046	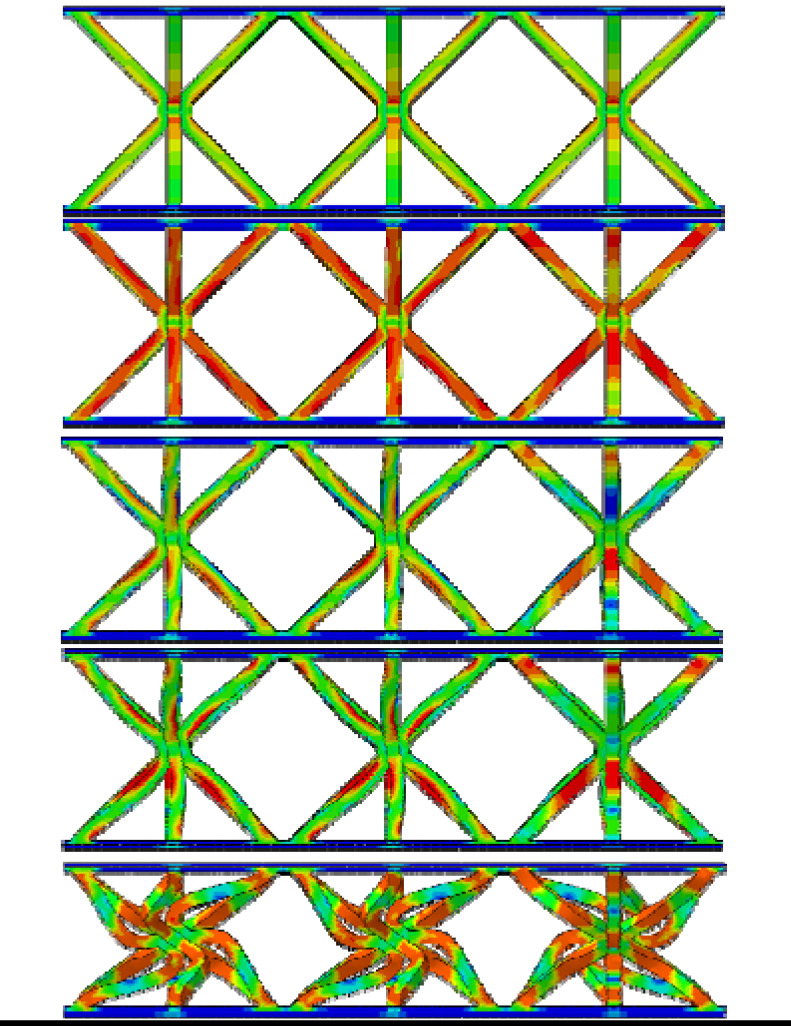	0.020	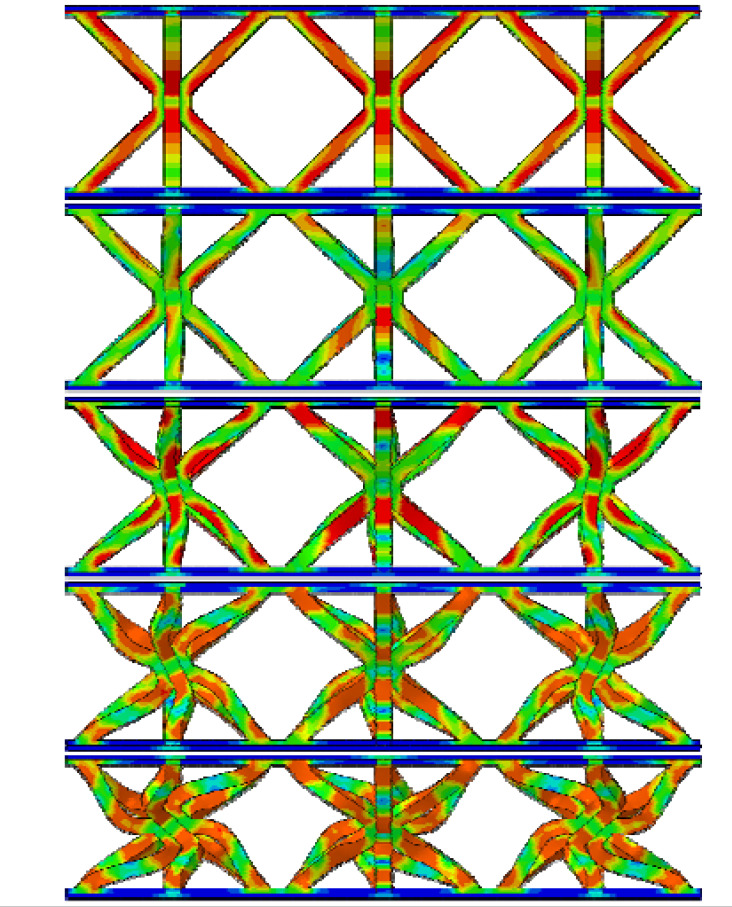
	0.025		0.065		0.040	
	0.050		0.120		0.090	
	0.140		0.180		0.164	
	0.220		0.245		0.310	

**Table 4:** Quasi-static plateau stress of the reinforced hourglass lattice structures.

$\bar{\rho }$	FE (MPa)	EX (MPa)	TP (MPa)
1.007%	0.41	0.47	0.44
1.76%	1.23	1.08	1.20
2.48%	2.25	2.24	2.05

**Table 5:** Structure parameters and in-plane compression performance.

Core Type	*h_c_* (mm)	*h_f_* (mm)	$\bar{\rho }$ (%)	*F* (N)	*W* (J)
Reinforced Hourglass	30	0.40	1.007	15,686	26,214
Hourglass			1.15	13,813	20,032
Pyramid			0.96	2958	7700

**Table 6:** In-plane compressive deformation process of three different structures. (The red circles and green arrows indicate the primary yielding locations of the face-sheet).

Structural Type	Reinforced Hourglass					Hourglass					Pyramid				
Deformation	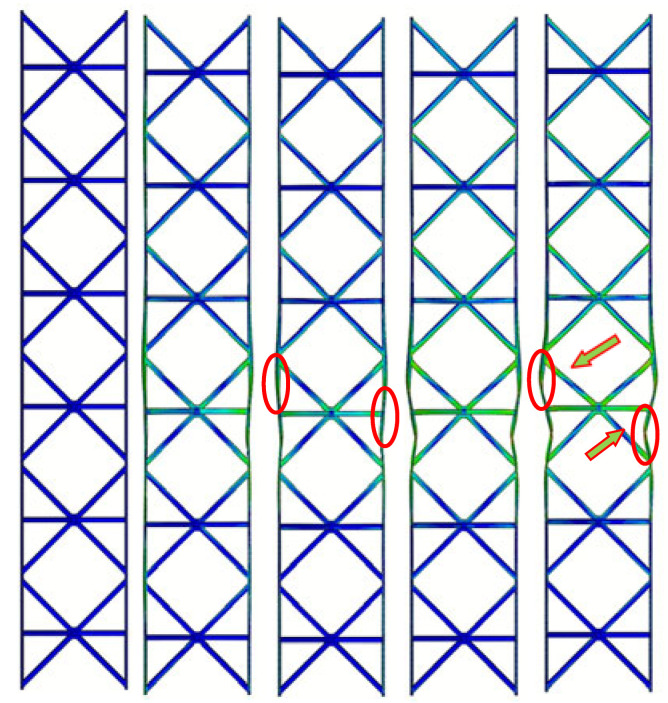					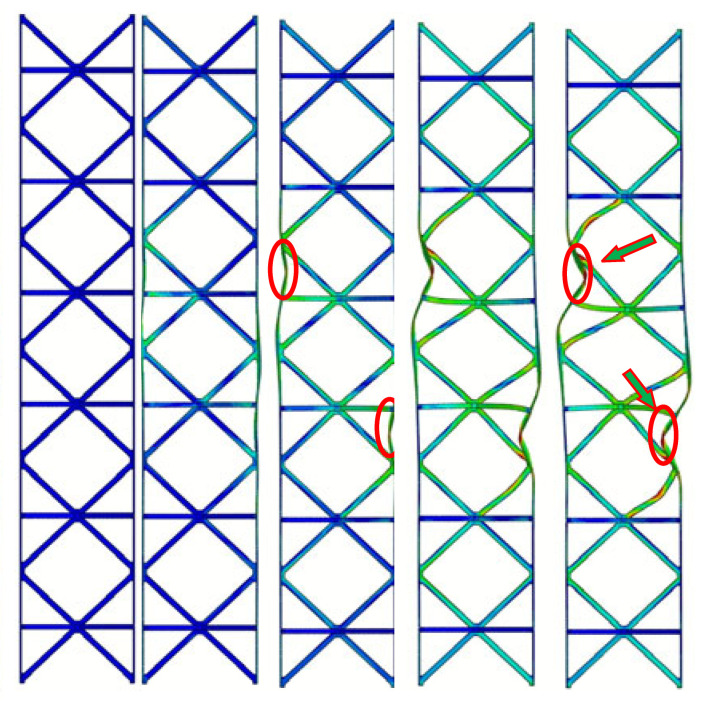					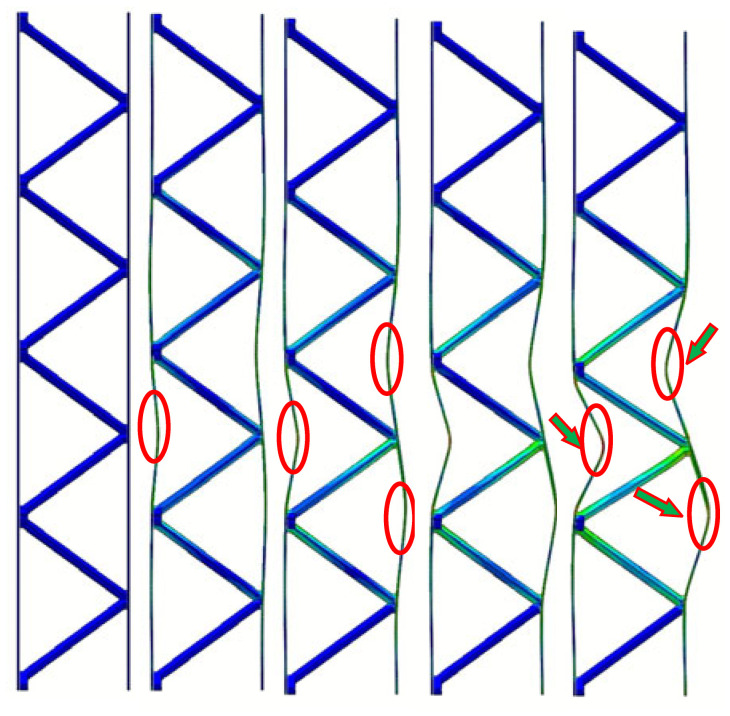				
Displacement (mm)	0.6	0.9	1.0	1.5	3.3	0.8	1.0	1.1	1.5	3.0	0.3	0.4	0.6	1.2	3.5

## Data Availability

The original contributions presented in this study are included in the article. Further inquiries can be directed at the corresponding authors.

## Abbreviations

The following abbreviations are used in this manuscript:

$\bar{\rho }$	Relative density
WEDM	Wire Electrical Discharge Machining
C3D8R	Continuum, 3-Dimensional, 8-node, Reduced integration
t/l	Aspect Ratio
*Mp*	Plastic bending moment
$\sigma_{s}$	Material stress
$\theta_{0}$,$\;\theta_{1}$	Deformation angles of the reinforced hourglass unit cell during compression
∆y	Axial displacement
$F_{p}$	Compressive force
$\sigma_{p}$	Theoretical plateau stresses
TP	Theoretical predictions
FE	Finite element
EX	Experimental
